# Cytotoxic Activity of Highly Purified Silver Nanoparticles Sol Against Cells of Human Immune System

**DOI:** 10.1007/s12010-015-1613-3

**Published:** 2015-04-23

**Authors:** Anna Barbasz, Magdalena Oćwieja, Jakub Barbasz

**Affiliations:** Institute of Biology, Pedagogical University of Cracow, Podchorążych 2, 30-084 Cracow, Poland; Jerzy Haber Institute of Catalysis and Surface Chemistry, Polish Academy of Sciences, Niezapominajek 8, 30-239 Cracow, Poland

**Keywords:** Silver nanoparticles, Tannic acid, Cytotoxicity, Granulocytes, Macrophages

## Abstract

The widespread use of silver nanoparticles (AgN) in the articles of common use justifies the need to investigate their effects on the human body. Nanosilver toxicity of highly purified, stable, and well-characterized Ag sol toward human immune cells at various differentiation stages has been studied. Human promyelocytic leukemia cells (HL-60) were differentiated to granulocytes using dimethyl sulfoxide and to macrophage-like cells by phorbol ester. Human monocytic cells (U-937) were differentiated to monocytes and macrophages by phorbol ester. In the presence of AgN, different changes of their survival time were observed depending on cell differentiation. Differentiated cells showed a significantly higher resistance than the non-differentiated cells, depending on the contact time and AgN concentration. In the presence of AgN at concentration of 25 mg/l, fraction of non-differentiated cells alive after 24 h was equal to 45 %; for granulocytes this number increased to 75 % and for macrophages to 65 %. The presence of AgN increases the levels of intracellular antioxidant —glutathione and of nitric oxide — one of inflammation mediators. By checking the effect caused by effluent obtained from AgN sol purification resulting at AgN sol purification, it was proved that cytotoxity should be attributed to the action of silver particles themselves.

## Introduction

Silver nanoparticles (AgN) are currently used in many areas of both science and everyday life. They are added to chemicals, food, cosmetics, clothing, and also to the range of household products. Nowadays, nanoparticles are predominantly used as antimicrobial [1], therapeutic [2] agents and also as fluorescent labels [3]. In medicine, AgN are exploited in drug delivery [4], molecular imaging [5], diagnostics, and treatment of cardiovascular diseases [6]. Thus, with an increase in nanoparticle applications, the exposure of living organisms to contact with them rises. Previously, AgN toxicity to numerous strains of bacteria, fungi, viruses, and algae was indicated [7]. The AgN interaction with bacteria consists in the release of silver ions that will result in the formation of reactive oxygen species (ROS) which further lead to cell membrane damage. However, many contradictory mechanisms of AgN action were reported [7]. AgN toxicity to fishes such as zebrafish [8, 9], to *Diptera* species [10] and to cell lines from mice [11], rats [12], and humans [13–18] was demonstrated. Studies on cells treated by silver nanoparticles showed the reduction of mitochondrion function, membrane damage, and oxidative stress causing cellular damage [19]. Unfortunately, “good” antibacterial properties of nanoparticles are in opposition to their potential toxicity to human cells and consequently to the entire human organism. This toxicity may be primarily associated with metallic nature of particles, resulting in changes of protein structure and activity leading to disorganization of cell functions [11, 20].

There are number of methods of preparation of silver nanoparticles: physical, physicochemical, and biological. Chemical approaches are the most popular for the production of nanoparticles. Biological methods are based on synthesis by microorganisms [21–24]. In current work, AgN were synthesized by chemical reduction of silver ions by tannic acid. This natural polyphenolic reducer belongs to the group of hydrolysable tannins, which contain glucose, esterified by gallic acid in central core [25]. Thanks to the specific structure, tannic acid has reducing and stabilizing properties, which causes its increasing use for the synthesis of silver [26, 27], gold [28, 29], and nickel [30] nanoparticles. Literature reports indicate that tannic acid exhibits natural antioxidant [31–35] and antiviral activity [36, 37]. As an antioxidant, tannic acid and its derivatives especially gallic acid and pyrogallol, by scavenging oxygen and oxygen-derived radicals, prevent lipid oxidation and radical-mediated DNA cleavage [32]. There are numerous reports indicating that tannic acid can inhibit the mutagenicity of certain mutagens [38, 39] and exert cancer chemopreventative activity in various animal models [40]. As is generally known, at low pH values, tannic acid exhibits weak reducing properties and only an increase of pH to high values (alkaline region) ensures an effective reduction of ions and nanoparticle formation [26]. Under mild basic conditions, tannic acid undergoes partial hydrolysis onto glucose and gallic acid [41]. Despite many postulated reaction mechanisms [26, 30], it is not clear whether tannic acid or products of its hydrolysis are relevant reducing agents. Taking into account that both gallic acid and glucose show comparatively poor stabilizing properties [26, 42], there is no doubt that the presence of unreacted molecules of tannic acid or the quinoid compounds with keto-enol systems generated during the oxidation reactions are responsible for the stability of the synthesized nanoparticles. On the other hand, as showed by Kim and coworkers, the processed tannic acid has stronger antioxidant capacity and antibacterial activity than freshly prepared solutions [37] because the mixtures, depending on the type of process (thermal or chemical hydrolysis), may contain different amounts of gallic acid, pyrogallol, or higher molecular weight keto-enol compounds [35]. In view of this silver nanoparticle, sol synthesized using tannic acid can have unique properties, as was shown in our previous studies where AgN sols obtained from different synthesis had different antibacterial activity against specially selected strains of *Escherichia coli* [43].

Earlier observations suggest that physicochemical properties of silver nanoparticle sols significantly influence their biological activity. The cytotoxicity of silver nanoparticles depends on factors such as particle size, shape, capping agent, and surface charge [44–46].

With a growing resistance of viruses, bacteria, and fungi to drug treatment, scientists tend to increase the use of nanosilver in medicine. Various studies have been done on the toxicity of silver nanoparticles relative to a range of organisms, but the exact mechanism of their action is still unclear. One of the main problems related to the synthesis of nanoparticles is the residue obtained after post reaction purification. Nanoparticles used in the current study were carefully purified and well characterized by physicochemical methods. This allowed to undertake a study on the effects of well-defined silver nanoparticles on cells of the human immune system not affected by the influence of the reactants remained after synthesis. The aim of the paper was to investigate the effect of silver nanoparticles on the functioning of human cell line HL-60 and U-937. The experiments were conducted on cells undifferentiated and differentiated to granulocytes and to monocytes and macrophages.

## Materials and Methods

### Materials

All chemical reagents used for synthesis of silver nanoparticles (silver nitrate, tannic acid, and ammonia (25 wt% NH_3_) were commercial products of Sigma-Aldrich and Avantor Performance Materials Poland S.A. (formerly POCH S.A.). Natural ruby mica sheets obtained from Continental Trade were used as a substrate for nanoparticle deposition. The pieces of mica were freshly cleaved into thin fragments of desired area. In order to promote silver particle deposition, the cationic polyelectrolyte, poly(allylamine hydrochloride) (PAH), having a molecular weight of 70 kDa (Polysciences) was used for modification of mica surface. Ultrapure water used throughout these investigations was obtained using the Milli-Q Elix&Simplicity 185 purification system from Millipore SA Molsheim, France. Dimethyl sulfoxide (DMSO) and 12-myristate-13-acetate (PMA) and all standard chemicals were purchased from Sigma (USA). RPMI 1640 medium, fetal bovine serum (FBS), and antibiotics were from CytoGen GmbH (Germany).

### Preparation of Silver Nanoparticles

Silver nanoparticle sols were prepared according to the modified Sivaraman’s method using tannic acid as a reducing agent [26]. Silver nitrate was dissolved in distilled water at room temperature to get silver ion concentration of 1 mM. Ten milliliters of 0.6 mM tannic acid aqueous solution (previously filtered through 0.22-μm Millipore filters) was added to the stirred silver precursor of volume 350 ml. Then, the pH value of the solution was adjusted with ammonia to the value of 9. The reaction mixture immediately became dark-yellow, but stirring was continued for 60 min.

To remove unreacted ions, obtained sol was washed with deionized water using a stirred membrane filtration cell (Millipore, model 8400) with a cellulose membrane (Millipore, NMWL: 100 kDa). The washing procedure was repeated until conductivity of the filtrate stabilized at 8–10 μS/cm and pH at 5.5–5.8.

### Nanoparticle Characterization Techniques

In order to achieve quantitative information about cytotoxicity of silver nanoparticles, obtained colloidal suspension was characterized using various physicochemical techniques. The silver nanoparticle concentration in the suspension was determined by a high-precision densiometer Anton Paar, type DMA5000M according to the procedure described previously [47].

UV-Vis spectra of silver nanoparticle sol and tannic acid solutions were recorded with the Shimadzu UV-1800 spectrometer using 1 cm path length quartz cell. Spectra were measured versus deionized water used as the reference sample.

The sizes and morphology of silver particles were determined by scanning electron microscopy (JEOL JSM-7500 F) working in transmission mode. Samples for this examination were prepared by dispersing a drop of the silver colloid on a copper grid, then covered by carbon film. Independently, atomic force microscopy (AFM) pictures of silver nanoparticles deposited on polyelectrolyte (PAH)-covered mica [42] were registered by the NT-MDT Solver Pro instrument equipped with the SMENA SFC050L scanning head. Imaging was done in semicontact mode using composite probes possessing a silicon body, polysillicon levers, and silicon high-resolution tips. The average values of the particle size, polydispersity, and stability of suspensions were determined by the dynamic light scattering (DLS) measurements (ZetaSizerNano ZS apparatus from Malvern Instruments). Electrophoretic mobility of nanoparticles at controlled conditions obtained from ZetaSizerNano ZS allowed to determine their zeta potential.

### Cell Cultures and Nanoparticle Treatment

Human promyelocytic cell line HL-60 (ATCC) was cultured in suspension in RPMI 1640 medium containing 10 % FBS and 0.01 % penicillin-streptomycin (full medium). Differentiation into macrophage-like cells was achieved by placing human promyelocytic leukemia cells (HL-60) in the phorbol-12-myristate-13-acetate at a dose 3 × 10^−9^ M in RPMI 1640 medium with 10 % FBS and 0.01 % penicillin-streptomycin and keeping cells in this mixture for 6 days [48]. The differentiation into granulocytes was realized by using 1.5 % dimethyl sulfoxide (DMSO) in RPMI 1640 with 10 % FBS and 0.01 % penicillin-streptomycin and holding cells in this for 6 days [48, 49].

Human histiocytic lymphoma cell line U-937 (ATCC) was cultured in suspension in RPMI 1640 containing 5 % FBS and 0.01 % penicillin-streptomycin. The activated macrophages were obtained by inducting the U-937 cells with 1 μM PMA for 24 h in the full medium.

Stock solution of silver nanoparticles (330 mg/l) and the effluent were diluted in RPMI 1640 medium to the required concentration. Sol of nanoparticles at appropriate concentration was added to the cultures to obtain defined final AgN level and incubated for 24, 48, and 72 h. To detect morphological changes of AgN-treated cells, pictures were observed under optical microscope.

AFM pictures of cells were obtained using the NT-MDT Ntegra Vita instrument with the scanning head and stage combined with Olympus IX71 inverted microscope. *Z*-axis was scanned by head and *X* and *Y* by the stage. AFM imaging was done in contact and semi contact mode. In C-AFM CSG01 probe, polysillicon levers and silicon high-resolution tips were applied. In semi contact mode, NSG03 tips were used. Scanned area was chosen on the basis of microscopic picture.

### Cell Viability Assay

The MTT tetrazolium salt colorimetric assay described by Mosmann [50] was used to detect cytotoxicity of nanoparticles. Cells were cultured in 24-well plates in an amount of 0.25 × 10^6^ HL-60 cells or 0.5 × 10^6^ U-937 cells per well in volume of 0.3 ml. After incubation with nanoparticles, 50 μl MTT solution (5 mg/ml) in sterile water was added to each well and left for 2-h incubation at 37 °C. Then, 0.3 ml of dimethyl sulfoxide (DMSO) was added to each well for 5 min. After centrifugation, the optical density of supernatant was read at 570 nm. Data were expressed as mean ± SD. The statistical analysis was performed by Duncan’s multiple range test, taking *p* < 0.05 using PC SAS 8.0

### Nitric Oxide Production

Cells (1 × 10^6^ cells/well) were treated with AgN to reach final volume of suspension equal to 0.5 ml and kept for 24, 48, or 72 h. After treatment, the supernatants were collected, centrifuged (1000 × *g*, 5 min), and stored at −20 °C. Nitric oxide (NO) production from AgN-treated cells was quantified spectrophotometrically using the Griess reagent (modified) (Sigma). The absorbance was measured at 540 nm, and the nitrite concentration was determined using calibration curve [51]. Data were expressed as mean ± SD. The statistical analysis was performed by Duncan’s multiple range test, taking *p* < 0.05 using PC SAS 8.0.

### Measurements of GSH

The intracellular reduced glutathione (GSH) level was determined by the reaction with 5,5′-dithiobis(2-nitrobenzoic acid) (DTNB)—the method of Ellman [52]. Cells (3 × 10^6^ cells/well) were treated with AgN in 24-well plate (1 ml final volume) for 24 h. Seventy-five microliters of 50 % trichloroethanoic acid was added and mixed for 30 s. The samples were centrifuged at 12,000 × *g* for 10 min at 4 °C, and the supernatants were transferred to a tube on ice. To 0.1 ml of the supernatants, the reaction mixture containing 0.8 ml of phosphate buffer (pH = 8.2) and 0.1 ml of 6 mM DTNB was added. Blank samples without supernatant were also prepared. The absorbance was measured at 412 nm, and the GSH concentration was determined from a calibration curve. Values are expressed as μmol GSH/mg protein. Total protein content was determined using the Bradford reagent. The protein concentration was calculated based on the standard curve made with BSA at a concentration range 0 to 1 mg/ml protein. Data were expressed as mean ± SD. The statistical analysis was performed by Duncan’s multiple range test, taking *p* < 0.05 using PC SAS 8.0

## Results

### Characterization of Nanoparticles

**Fig. 1:**
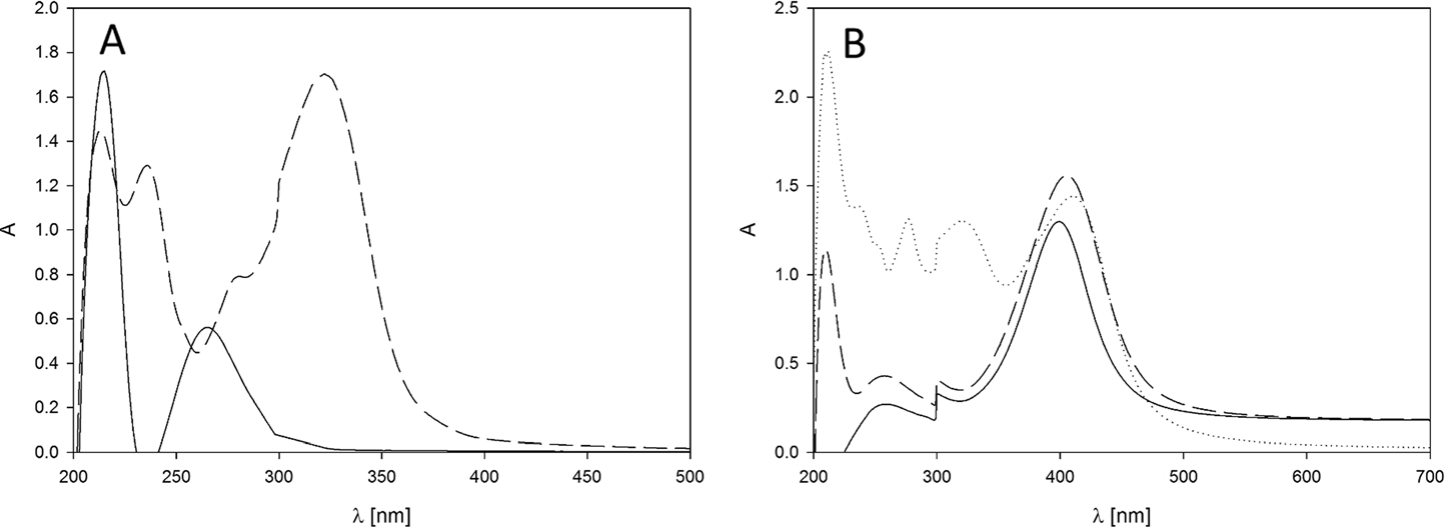
UV-Vis spectra of: **a** tannic acid solution (concentration 25 mg/l): *solid line* at pH 4.8 and *dash line* pH 9 and **b** silver nanoparticle sol: *dotted line* after addition of aqua ammonia to reach pH 9, *dash line* 1 h after synthesis, and *solid line* after purification by membrane filtration

Synthesis of silver nanoparticles was monitored using spectrophotometric method. In order to determine the promising composition of the reducing mixture necessary for the preparation of silver sol, the UV-Vis spectra of freshly prepared tannic acid solution at natural pH 4.8 and the solution at pH 9 obtained by addition of aqua ammonia were recorded. At natural pH, the absorption spectrum of aqueous solution of tannic acid exhibits two peaks at 214 and 271 nm (Fig. 1a) in accordance with literature data [53]. As can be seen, at pH 9 (Fig. 1a), the position of peaks is significantly shifted to higher wavelengths and their intensity decreased. Moreover, in the spectrum, the new peaks appear at 235 and 323 nm. According to the literature data, the presence of two isosbestic points in the spectrum supports the assumption that at least two different forms of tannic acid are of the phenolate type [53]. As can be noticed in Fig. 1b, these forms of tannic acid are still present in the initial step of silver nanoparticle formation. With time, their concentration in the mixture decreases, and after 1 h in the spectrum of reaction mixture, three peaks at 210, 255, and 401 nm are observed. The last absorbance peak is attributed to the plasmon excitation of silver nanoparticles. The positions of the other peaks correspond to the characteristic bands neither tannic nor gallic acids [54] but probably to the oxidized forms of phenolic compounds. As shown in the Fig. 1b, careful cleaning by membrane filtration allowed to remove the excess of these compounds from the post reaction mixture.

After filtration, the weight concentration of the silver nanoparticles in the sol was precisely determined. Density of the silver sol and the filtrate solution acquired from membrane filtration were measured using densitometer DMA 5000 M. Concentration of silver particles was calculated from formula described in detail in our previous work [47]. Concentration of silver particles in purified suspension amounted to 330 mg/l.

**Fig. 2:**
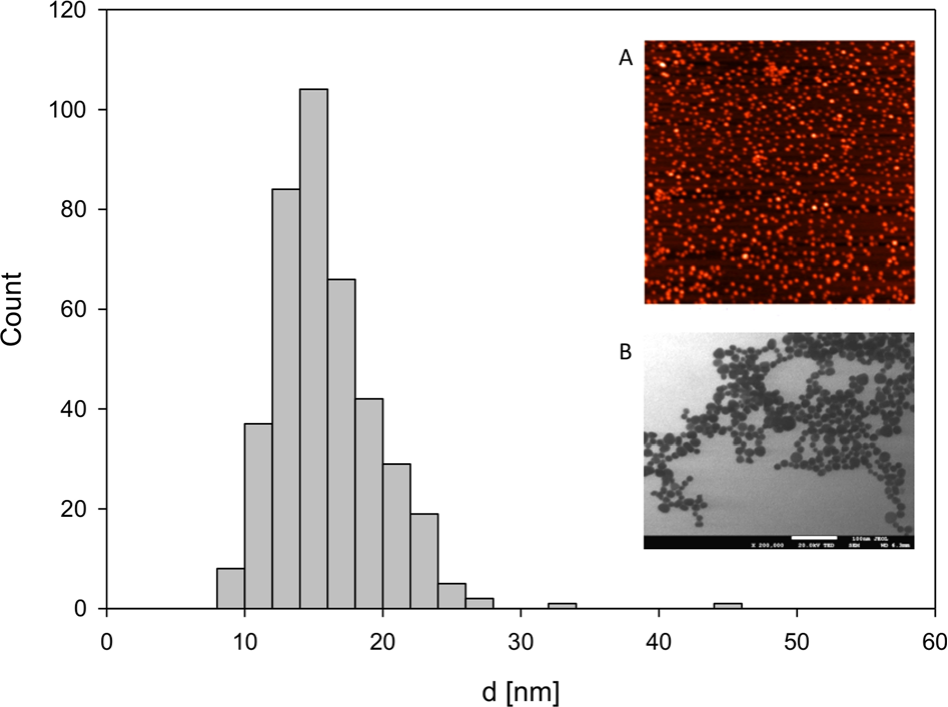
Presentation of obtained nanoparticles: main plot—the size distribution of nanoparticles obtained from TEM, *insets*: *a* AFM image of monolayers of nanoparticles deposited on PAH-covered mica and *b* TEM micrograph of nanoparticles deposited on copper grid

The size distribution and shape of nanoparticles were determined using microscopic methods: AFM and transmission electron microscopy (TEM). As can be noticed in the AFM image and TEM micrograph (Fig. 2 insets a, b), the nanoparticles exhibit nearly spherical shape and small size. Size of a single nanoparticle was defined as an average of the two maximal diameters perpendicular to each other and from the cross-section area determined by MultiScan Base software. The distribution of such defined particle sizes was presented by histogram showing the number of particles in the given diameter range (Fig. 2). Based on the obtained histogram, it was found that an average size of nanoparticles equals to 17 ± 5 nm.

The size of nanoparticles in sol was measured by the light scattering method (DLS). Knowing that the nanoparticles exhibit spherical shape, the size of particles in the bulk sol was calculated from the values of diffusion coefficients using the Stokes-Einstein relationship:

1 $$ {d}_H=\frac{kT}{3\pi \eta D} $$

where *d*_*H*_ is the hydrodynamic diameter, *k* is the Boltzman constant, *T* is the absolute temperature, and η is the dynamic viscosity of the solution.

The advantage of using the hydrodynamic diameter over the diffusion coefficient is that it is independent of temperature and liquid viscosity, so it is an appropriate parameter for analyzing suspension aggregation phenomena. It is well known that the activity of silver nanoparticles in biological systems strongly depends on the sol stability. Therefore, the dynamic light scattering measurements were applied for determination sol stability under various conditions: pH and ionic strength adjusted by the addition of appropriate amount of hydrochloric acid, sodium hydroxide, and sodium chloride.

**Fig. 3:**
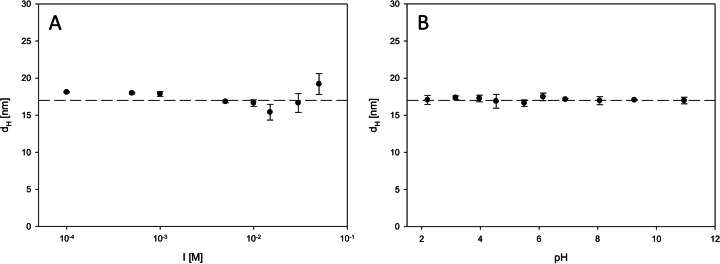
The dependence of the hydrodynamic diameter of nanoparticles (obtained from dynamic light scattering) on **a** ionic strength at pH 5.5 and **b** pH at *I* = 0.01 M NaCl. Measurement conditions: particle concentration in sol *c*_p_ = 25 mg/l, *T* = 298 K. The *dashed lines* for leading the eye

The dependence of hydrodynamic diameter on ionic strength in the range 10^−4^– 0.05 M NaCl at pH 5.5 is shown in Fig. 3a. The effect of pH on *d*_*H*_ at a fixed ionic strength equal to 0.01 M is shown in Fig. 3b. The obtained results indicate that nanoparticles’ hydrodynamic diameters are practically constant what means that the sol is stable in the wide range of ionic strengths and pH. It is worth to mention that an average size of nanoparticles determined by DLS technique strictly corresponds to the results obtained from microscopic measurements.

The physicochemical characteristics of the silver particle suspension were supplemented by the electrophoretic mobility (μ_*e*_) measurements performed by the method described in our previous work [47]. Knowing the electrophoretic mobility for given conditions, one can calculate the particle zeta potential using equation:

2 $$ {\zeta}_p=\frac{\eta }{\varepsilon f\left(\kappa {d}_p\right)}{\mu}_e $$

where ζ_*p*_ is the zeta potential of particles, ε is the electric permittivity of the solution, *f*(κ*d*_*p*_) is the correction function of the dimensionless parameter κ*d*_*p*_, and ${\kappa}^{-1}={\left(\frac{\varepsilon \kern0.1em kT}{2{e}^2I}\right)}^{1/2}$ is the thickness of the electric double layer. For thin double layers (κ*d*_*p*_ > 100), *f*(κ*d*_*p*_) approaches unity (Smoluchowski’s approximation), and for thick double layers (κ*d*_*p*_ close to 1), *f*(κ*d*_*p*_) approaches 2/3 (Hückel’ approximation).

**Fig. 4:**
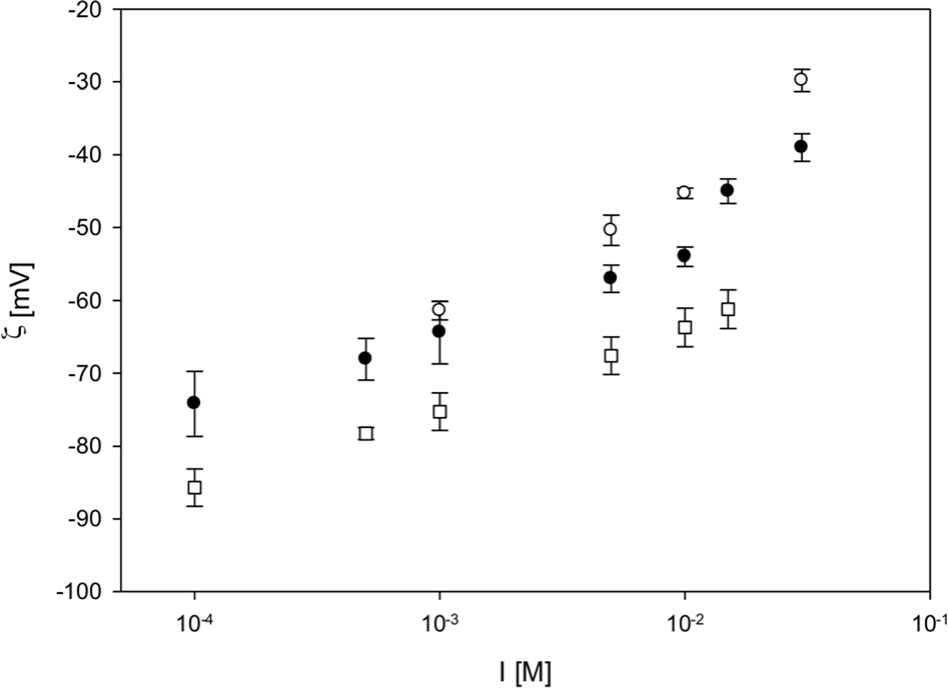
The dependence of the zeta potential of silver nanoparticles on ionic strength for pH 3 (*empty circle*), pH 5.5 (*filled circle*), and pH 9 (*square*). Measurement conditions: particle concentration *c*_p_ = 25 mg/l, *T* = 298 K

As electrostatic interactions play an important role in stability of systems in polar media, a number of measurements of electrophoretic mobility of silver particles were carried out for various solution’s ionic strength and pH. The obtained results are shown in the Fig. 4. As can be seen, the zeta potential of silver particles assumed high negative values for the entire range of ionic strength and pH. The values of zeta potential increased slightly with ionic strength and attained the less negative values for the lowest pH. The results of hydrodynamic diameter and zeta potential of silver particles in purified sols confirm that the obtained system is stable in the wide range of pH and ionic strength. Thus above, thorough physicochemical analysis proved that Ag nanoparticles used for the biological assays represent single objects (not aggregates).

### Cytotoxicity in Cultured HL-60 and U-937 Cells

**Fig. 5:**
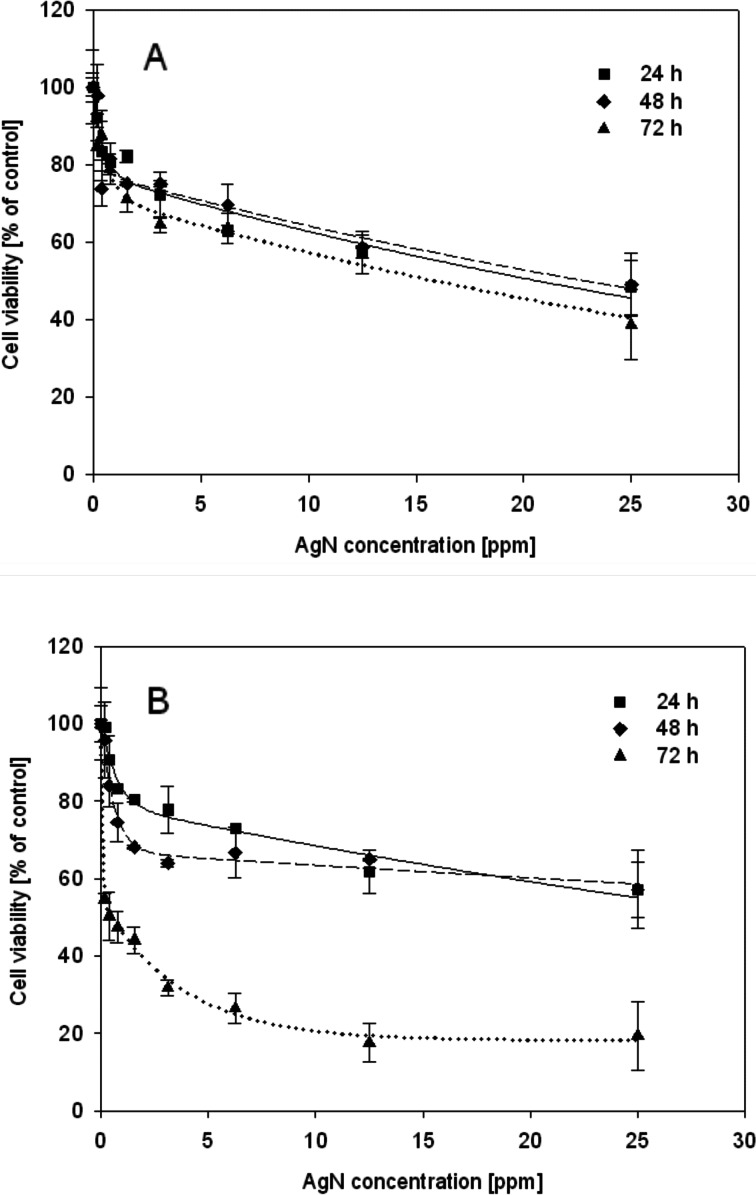
The dependence of cell viability on AgN concentration. Cell viability was determined by using the MTT assay. Viability of the treated group: HL-60 (**a**) and U-937 cells (**b**) was expressed as a percentage of the control group (cells not treated by AgN). Cells were incubated with AgN for 24, 48, and 72 h. Data points are means ± standard deviations (*n* = 6) from a representative experiment

**Fig. 6:**
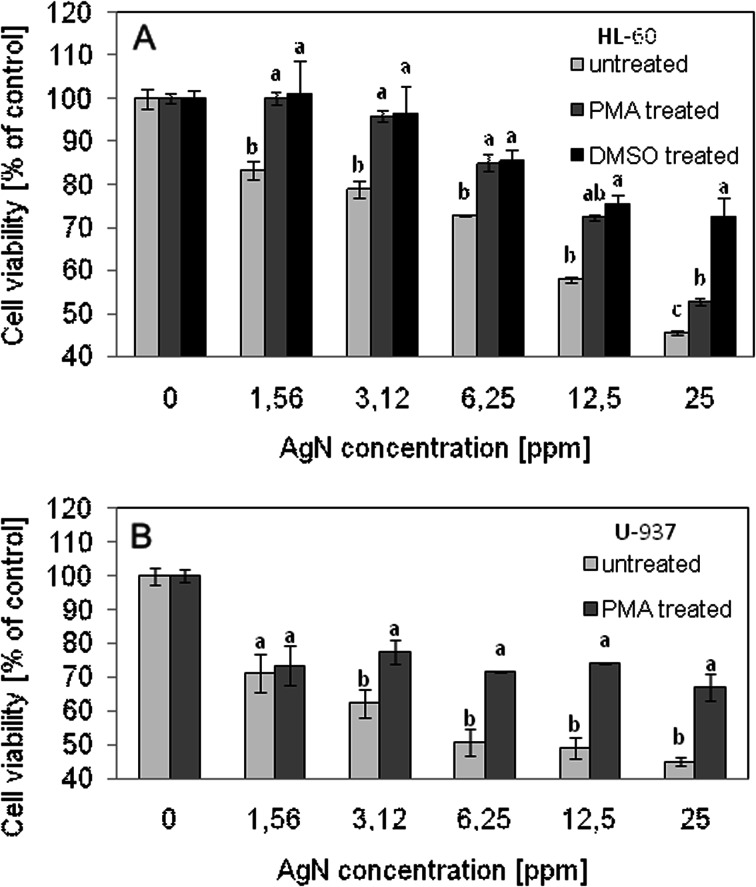
Effect of cell differentiation on their survival. **a** HL-60 cells differentiated for 6 days using 3 nM PMA or 1.5 % DMSO, subsequently 24 h incubated with AgN. **b** U-937 cells activated 1 day by 1 μM PMA and incubated with AgN for 24 h. Data points are means ± standard deviations (*n* = 6) from a representative experiment. *Different letters* indicate significant (*p* < 0.05) differences between treatments

As a probe for cell viability testing, MTT assay was used. Viability defined as a fraction of cells alive after defined time of both cultured cells decreased with an increase of AgN concentration. Viability of HL-60 cells diminished to about 60 % in the presence of AgN at concentration of 25 mg/l regardless of the length of the exposure time. At the same silver sol concentration (AgN 25 mg/l), viability of U-937 cells decreased to about 70 % after 24 and 48 h and to about 20 % after 72 h (Fig. 5). Cell lines selected for these studies are characterized by the ability to differentiate into specialized cells of the immune system. Cell survival was also tested at various stages of cell differentiation, i.e., for untreated cells as well as cells differentiated to granulocytes and monocytes and macrophages (cell line HL-60) or activated monocytes and macrophages (line U-937). As seen in Fig. 6, differences in the survival were observed for the cells treated with PMA and DMSO. At AgN concentration of 25 mg/l, survival of HL-60 cells decreased to 55 %, whereas fraction of alive macrophage-differentiated cells was equal to 45 % and granulocyte-differentiated cells was only 25 % relative to control (cells untreated by AgN).

**Fig. 7:**
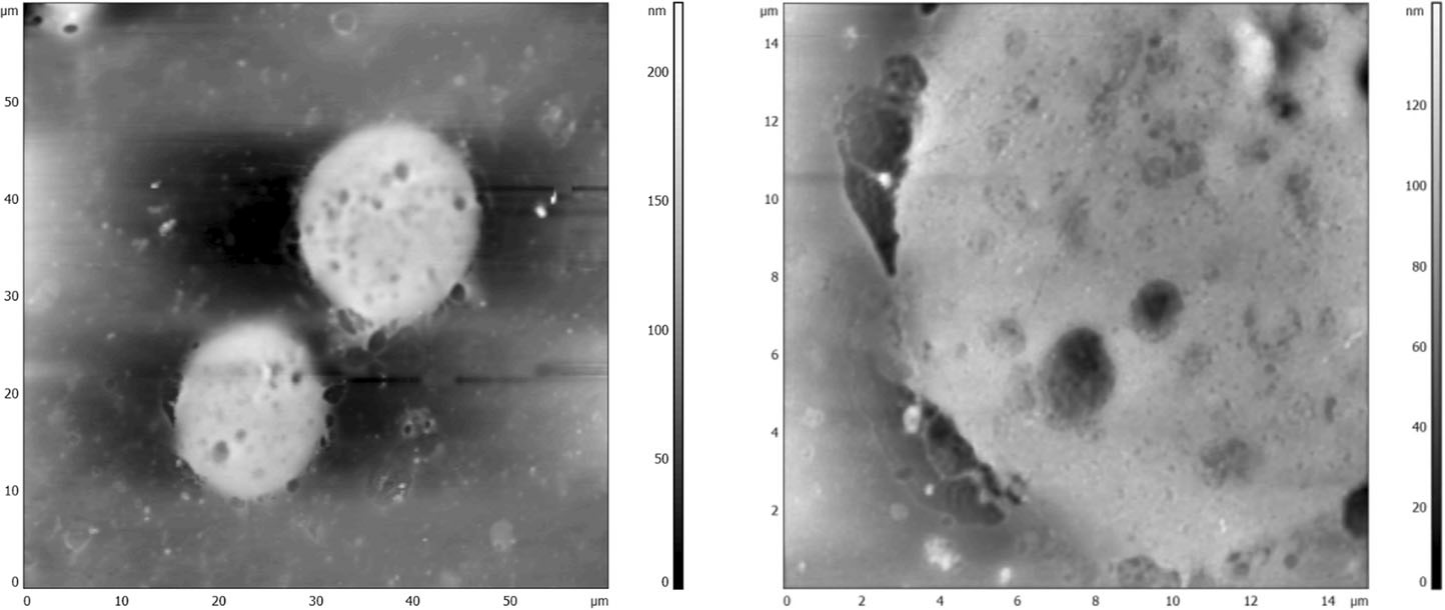
AFM image of U-937 cells after 24 h contact with silver nanoparticles. The areas of scans: *right *— 60 μm × 60 μm, *left *— 15 μm × 15 μm. Image was taken in contact mode in dry conditions

The cells after treatment with silver nanoparticles were observed using AFM techniques. Holes (700 –2000 nm diameter) on the surface of U-937 cells were observed (Fig. 7). Such holes were not present on surfaces of not-treated cells.

### The Level of NO

**Fig. 8:**
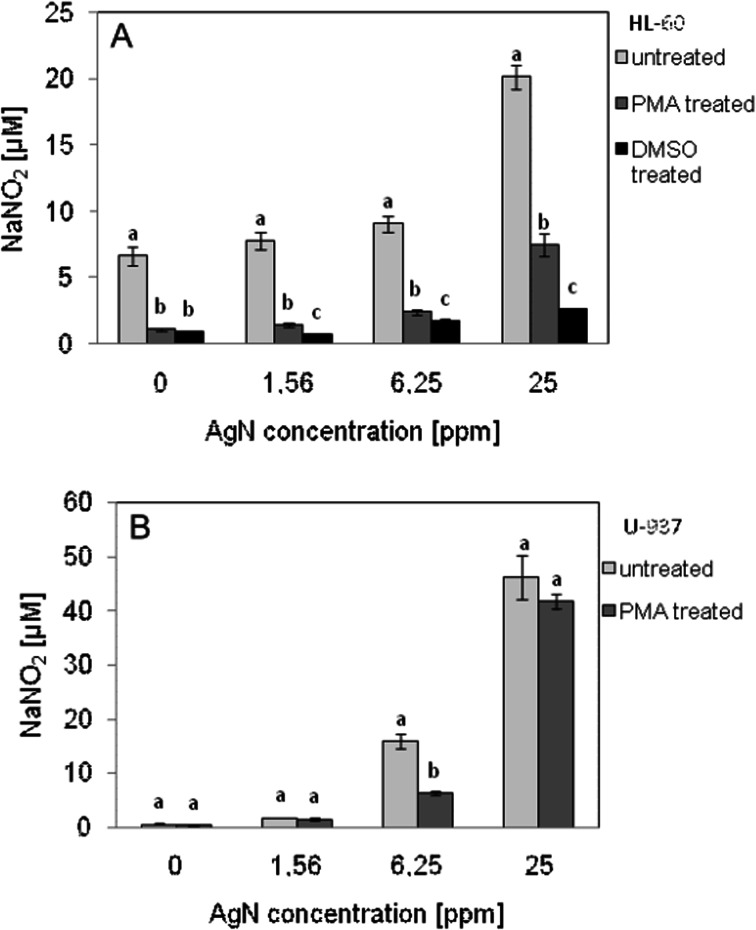
Level of NO secreted by HL-60 cells (**a**) or U-937 cells (**b**) after contact with AgN. NO production was quantified spectrophotometrically using the Griess reagent. Cells were treated with AgN with the indicated concentration for 24 h. Data points are means ± standard deviations (*n* = 6) from a representative experiment. *Different letters* indicate significant (*p* < 0.05) differences between treatments

To investigate the influence of AgN on inflammation, the level of secreted NO, acting as a second messenger in inflammatory signaling, was measured. As shown in Fig. 8, NO secretion was increased for both cell lines in the presence of AgN. Almost threefold increase of NO level was observed in the case of undifferentiated cells of HL-60 line and up to sevenfold increase for cells differentiated by phorbol ester after contacting with AgN sol of concentration 25 mg/l. U-937 cells both native and treated with PMA respond to AgN sol of 25 mg/l concentration by tenfold increase in the level of NO.

### The Level of Intracellular GSH

**Fig. 9:**
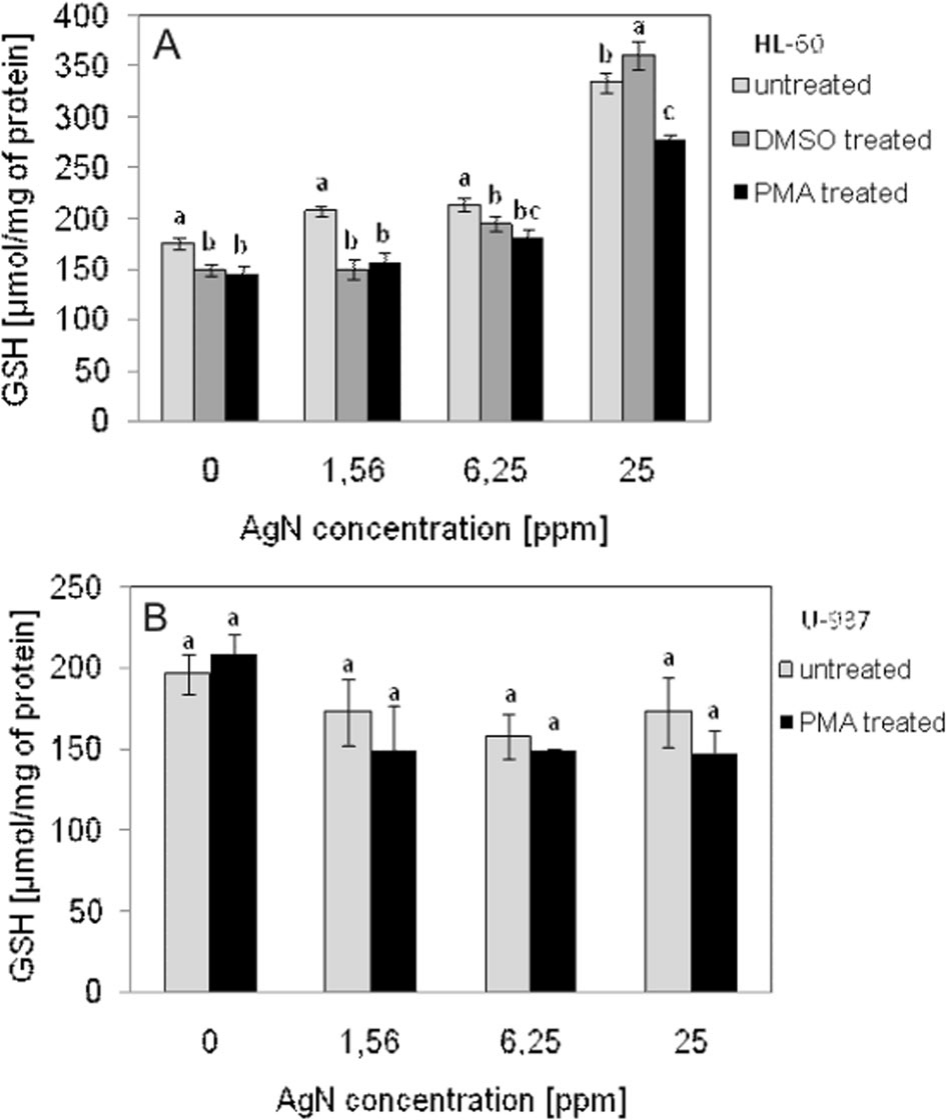
Concentration of intracellular GSH in HL-60 (**a**) or U-937 (**b**) cells treated for 24 h with AgN. Data points are means ± standard deviations (*n* = 6) from a representative experiment. *Different letters* indicate significant (*p* < 0.05) differences between treatments

For cell line HL-60 regardless of differentiation, twofold increase in intracellular glutathione content was observed after treatment by AgN at concentration of 25 mg/l. For the cell line U-937, the level of intracellular GSH was increased in the not-differentiated cells while in the cells activated with PMA, GSH level declined after contact with AgN sol at concentration of 25 mg/l (Fig. 9).

## Discussion

In these studies, a stable, highly purified silver sol containing particles of defined sizes, shape, and charge acquired by synthesis based on chemical reduction by organic phenolic compound (tannic acid) was used. Many publications have shown size-dependent cytotoxicity of nanosilver [44, 55–58]. When a particle size decreases, the surface area increases thereby increasing the number of silver atoms exposed to the external environment. Those atoms are available for all biochemical reactions and interactions with cells. The shape of the silver nanoparticles is also relevant for their bioactivity; truncated triangular nanoplates exhibit stronger antibacterial activity than spherical silver nanoparticles [47, 58]. As showed by Park et al. [59, 60], size of silver nanoparticles is of crucial importance in the development of the immune response. These authors treated human macrophages (U-937) by silver nanoparticles of sizes 4, 20, and 70 nm. It was shown that the smallest nanoparticles had the greatest potential proinflammatory ability (by inducing oxidative stress and releasing cytokine). Following these reports, particles used in the studies (17 nm) are close to the range where the size effect may be to neglect.

In the current work, the impact of silver nanoparticles on two human cell lines of immune system was determined. Immune cells present in the body ensure its protection against infections caused by microorganisms or foreign particles such as metal nanoparticles. The model line of HL-60 promyelocytic cells used for the research is non-adherent under standard culture conditions. Characteristic of HL-60 cells is their ability to differentiate into several types of cells of the myelomonocytic lineage. Various factors cause the differentiation of cells into four types: granulocytes, monocytes, macrophage-like cells, and eosinophils [49]. In the studies, two ways of differentiation were chosen: to monocytes and macrophages (by application of 3 nM PMA, differentiation occurring within 6 days) and to granulocytes (by addition of 1.5 % DMSO, differentiated product appearing after 6 days) [48]. The addition of polar-planer compound such as DMSO results in significant changes of morphological, functional, enzymatic, and surface membrane antigen’ characteristics of cells with consequent formation of mature granulocytes [49]. Cells of HL-60 line, differentiated to neutrophil-like cells, posses granulocyte’ nuclei, disintegrating into lobes. These cells exhibit an increased expression of CD11b antigen, ability to reduce NBT, and are able to phagocytosis [61]. Differentiation in vitro beside changes in cell functions causes significant modifications in the composition of membrane lipids and cell membrane fluidity [62].

Human monocytic cells U-937 by treatment with phorbol esters differentiate into monocytes and macrophages [63, 64]. After differentiation, they contain non-specific esterase, β-glucuronidase, elastase and secreted into the medium lysozyme. On the surface of U-937, Fc receptors, C3, and chemotactic peptides specific for monocytes are located [64]. Production of reactive oxygen species is possible only when the cells are activated with PMA [64]. In accordance with literature reports, cells of U-937 line after activation with phorbol esters are capable of production of reactive oxygen species. Incubation with PMA results in changes in the production of reactive oxygen species and the specific membrane receptors, affects the phagocytosis or chemotaxis [64, 65]. It is a feature of this cell line. Production of H_2_O_2_ and O_2_•^−^ by U-937 cells was studied in great detail. The ability to produce reactive oxygen species in this case is a measure of the cell maturity. The percentage of NBT-positive cells increased significantly after treatment with agents such as TPA, DMSO, LK, RA, VD3, and PMA [64, 65]. Under such conditions, the specific membrane receptors appear which affect phagocytosis and chemotaxis [65].

Nowadays, the opportunity of organisms to come into contact with silver nanoparticles is very large. AgN are used in medicine and are present in cosmetics and even textiles [66, 67]. Studies on biological activity versus human cells demonstrated that AgN induce cell necrosis or apoptosis of several cell types such as macrophages [68, 69]. In 2010, Park and coworkers proposed the “Trojan-horse mechanism” of AgN cytotoxicity to mousé peritoneal monocytes (RAW 264.7) [68, 69]. After exposure to AgN (commercially produced), the cell viability decreased in time- and concentration-dependent manner. Phagocytosis of AgN completely blocks cell cycle in the S-phase and stimulates inflammatory signaling through generation of reactive oxygen species, followed by induced secretion of TNF-α. A decrease in viability was also observed for liver cells and neurons treated with AgN [12, 70].

To detect the cellular response to the toxicant, the cell viability was determined. The fraction of alive HL-60 cells decreased proportionally to AgN concentration being independent on contact time, whereas viability of U-937 cells was dependent on both: concentration and time. Significant differences in cell survival were observed in the case of differentiated cells. For both cell lines, an increased survival of specialized cells was observed.

It is well known that silver nanoparticles induce toxicity due to promotion of oxidative stress and thus generation of ROS. In the case of bacteria, the following mechanisms of nanosilver action was proposed: (1) the initial uptake of silver ions which results in interruption of ATP production and retention of DNA replication, (2) the generation of ROS by the joint action of nanoparticles and silver ions, and (3) damage of cell membranes. The interaction of AgN with the surface of bacteria results in the cell damage and penetration of silver particles into the cell interior. The holes in the cell membranes were observed in case of the *E. coli* cells after contact with AgN [71–73]. Similar findings were reported for the surfaces of bacteria such as *Vibrio cholerea*, *Pseudomonas aeruginosa*, or *Salmonella typhus*, but only nanoparticles with diameters less than 10 nm adhered to the membrane and penetrated the inside of the bacterium [58]. Holes of diameter about 700–2000 nm on the surface of cells after contact with 17 nm nanosilver particles were observed in present studies by using AFM technique. One can conclude that the mechanism of the cell membrane destruction is similar for both bacterial and human cells.

It was found that regardless of cell types and differentiation, their contact with filtrate diluted the same way as was done for AgN sol caused the decrease of viability by 20 % (i.e., to the value of 80 % in comparison to non-treated cells). This shows that the toxic effect of AgN sol originates primarily from silver particles. This conclusion about AgN bioactivity could be reached thanks to the use in the experiments the reaction product carefully purified and well characterized.

NO has many biological activities, both in physiological and pathophysiological processes. NO acts as a mediator of inflammation. A marked increase in the nitrogen oxide content in the tested cell suspensions after treatment with AgN was observed. In the case of macrophages (differentiated HL-60 cells), NO level growth was nearly sevenfold at a AgN concentration of 25 mg/l. All reported experiments were also performed when treating the cells by filtrate obtained from AgN sol purification (results not shown). The results obtained showed that an increase of NO content was not observed indicating that the effluent did not activate immune cells. In view of the use of tannic acid in the synthesis of AgN, tests for the tannin presence in silver sol and in effluent were carried out. In the silver sol, tannin content, if any, was below detection limit of applied analytical methods. In effluent, the concentration of the tannin in effluent was found at 15 ppm. The obtained results allow concluding that AgN particles themselves cause an activation of the cells and induction of inflammation.

To investigate the correlation between oxidative stress and cytotoxicity induced by AgN, intracellular GSH was measured. The GSH acts as antioxidant. The increased concentration of ROS results in an increase in transcription of proinflammatory cytokines leading to apoptosis [74, 75]. In the case of HL-60 cell lines, intracellular GSH level increased almost twice similarly for native and differentiated cells treated by AgN. The effect of AgN on growth of intracellular glutathione content was also reported in literature for liver cells [19, 76]. Intracellular glutathione acts as a scavenger of ROS appearing in the cells under stress conditions. It is therefore possible that increased GSH content observed in the studies is associated with the initial mobilization of cells to defend against cytotoxic effects caused by nanosilver particles. Longer than 24 h time of incubation with AgN (applied in this research) may probably result in a reduction in the content of GSH in cells as was observed for murine macrophage RAW 264.7 [68, 69]. In the case of U-937 cells, their contact with AgN at studied concentrations practically did not influence the GSH amount.

As cancer cells have limited ability for differentiation by decreasing viability of non-differentiated cells (as it was found in presented experiments), nanosilver particles may be considered as a support for anticancer therapy. Currently, nanosilver particles are used mainly for imaging tumors in vivo and in therapy of few cancer types. For application of AgN in therapy of breast cancer, it is necessary to use samples containing particles of appropriate physical and chemical parameters. Therefore, the exact physicochemical characterization of nanoparticles as done in this work is essential for their potential application in medicine. Systems containing Ag particles used as carriers of bioactive factors can become a great weapon in the fight against cancer-related diseases [77, 78]. It is still very difficult to gain a control on toxic and beneficial effects of silver nanoparticles. Therefore, it is extremely important to separate the effects coming from the particles themselves and all other components of post reaction mixture.

Despite the ubiquitous presence of silver nanoparticles, the effect of AgN on the human immune system is still not well investigated [79]. Quantitative determination of the impact of AgN on human immune cell lines provides database that allows for developing a balance between cytotoxicity and the beneficial effects of silver nanoparticles.
